# Comparative analyses of chloroplast genome data representing nine green algae in Sphaeropleales (Chlorophyceae, Chlorophyta)

**DOI:** 10.1016/j.dib.2016.03.014

**Published:** 2016-03-09

**Authors:** Karolina Fučíková, Louise A. Lewis, Paul O. Lewis

**Affiliations:** Department of Ecology and Evolutionary Biology, University of Connecticut, Storrs, CT, USA

## Abstract

The chloroplast genomes of green algae are highly variable in their architecture. In this article we summarize gene content across newly obtained and published chloroplast genomes in Chlorophyceae, including new data from nine of species in Sphaeropleales (Chlorophyceae, Chlorophyta). We present genome architecture information, including genome synteny analysis across two groups of species. Also, we provide a phylogenetic tree obtained from analysis of gene order data for species in Chlorophyceae with fully sequenced chloroplast genomes. Further analyses and interpretation of the data can be found in “Chloroplast phylogenomic data from the green algal order Sphaeropleales (Chlorophyceae, Chlorophyta) reveal complex patterns of sequence evolution” (Fučíková et al., In review) [1].

**Specifications Table**

**Table:** 

Subject area	*Biology*
More specific subject area	*Phylogenomics*
Type of data	*Table, Figures, text file, tree file*
How data were acquired	*Phylogenetic analysis of gene order in Badger, Mauve analysis, Geneious genome map*
Data format	*Analyzed*
Experimental factors	*Genomic data were collected using Illumina HiSeq and annotated in Geneious*
Experimental features	*Gene order data analyzed in Badger, Synteny maps in Mauve plugin in Geneious*
Data source location	*Storrs, CT, U.S.A.*
Data accessibility	*Data is within this article and at NCBI:*http://www.ncbi.nlm.nih.gov*accessions GenBank: KT199248, GenBank: KT199249, GenBank: KT199250, GenBank: KT199251, GenBank: KT199252, GenBank: KT199253, GenBank: KT199254, GenBank: KT199255, GenBank: KT199256*

**Value of the data**

• Chloroplast genomes of green algae in the order Sphaeropleales are currently sparsely studied, thus new data from nine additional species expands knowledge of the structural variation within this order of algae.
• Table summarizes the features present in chloroplast genomes of green algae in Sphaeropleales, useful for comparison to other species in future analyses.
• Figures of nine assembled chloroplast genomes of green algae illustrate the features and their arrangements in these species.
• New gene order data can be used in future phylogenetic analyses that include information from additional species.

## 1 Data

**Fig. 1:**
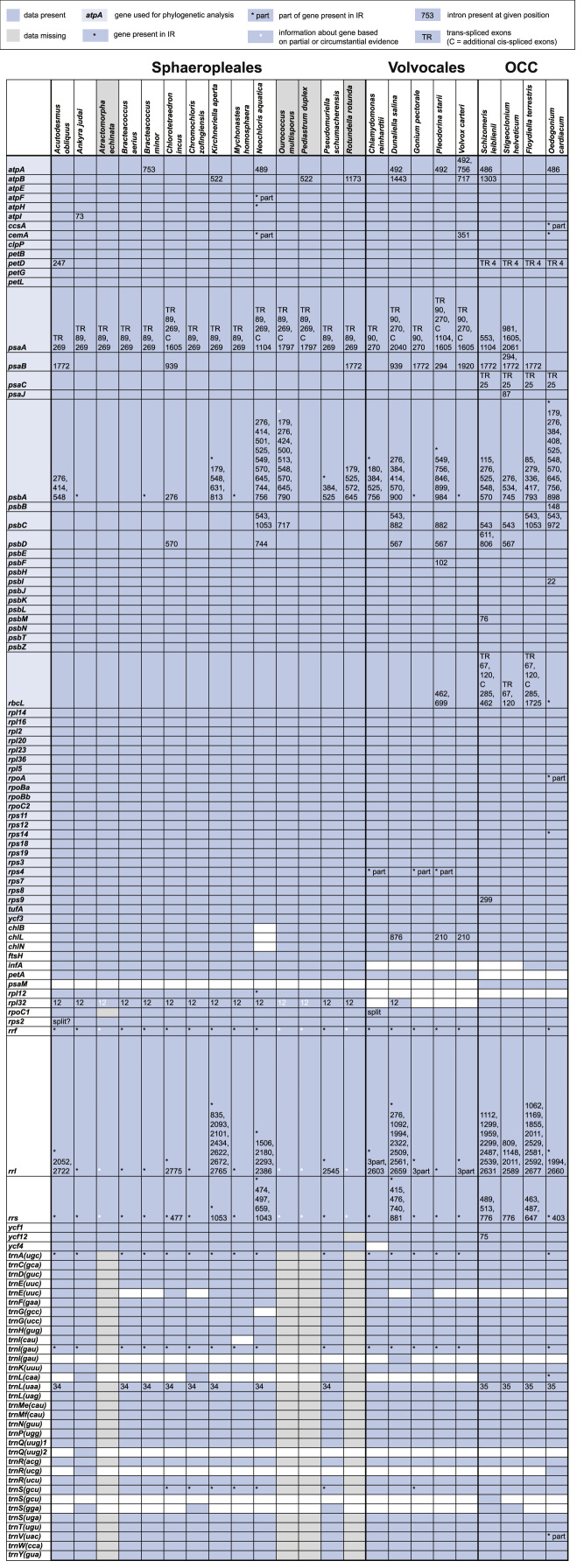
Comparison of gene presence and intron content in chloroplast genomes of algae in Chlorophyceae.

**Fig. 2:**
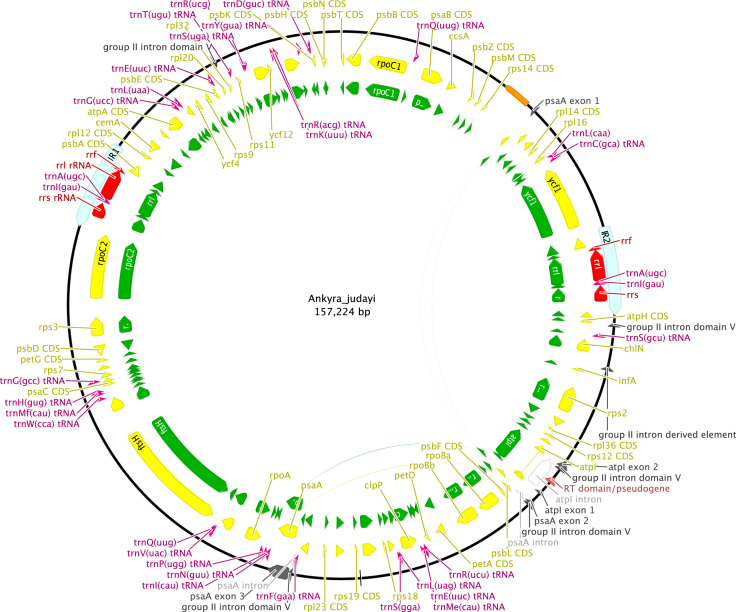
Schematic map of the chloroplast genome of *Ankyra judayi* (KT199255, SAG 17.84).

**Fig. 3:**
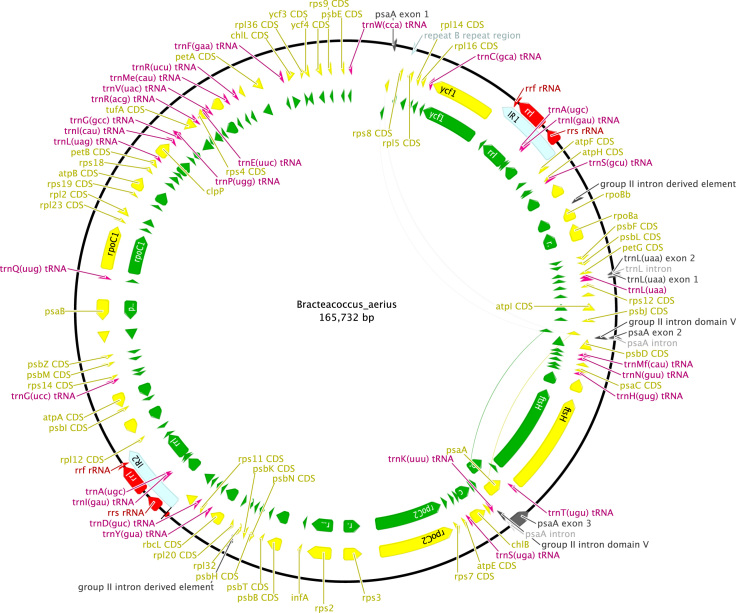
Schematic map of the chloroplast genome of *Bracteacoccus aerius* (KT199254, UTEX 1250).

**Fig. 4:**
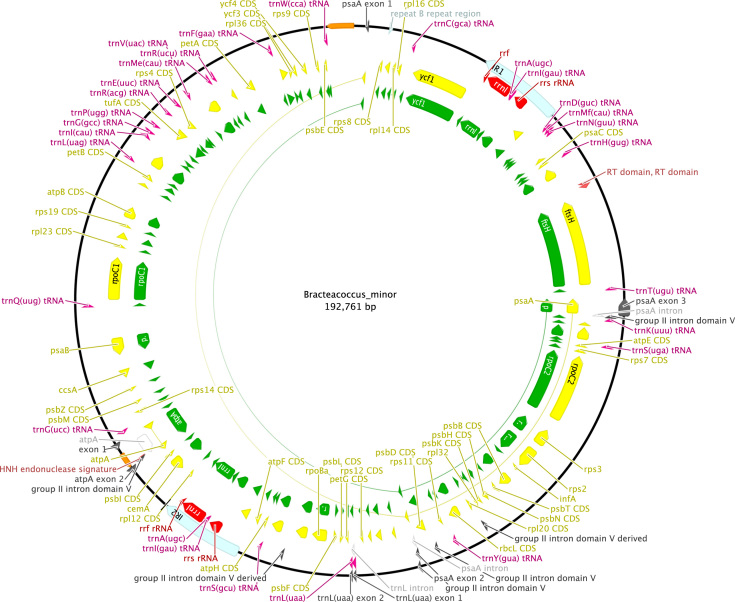
Schematic map of the chloroplast genome of *Bracteacoccus minor* (KT199253, UTEX B 66).

**Fig. 5:**
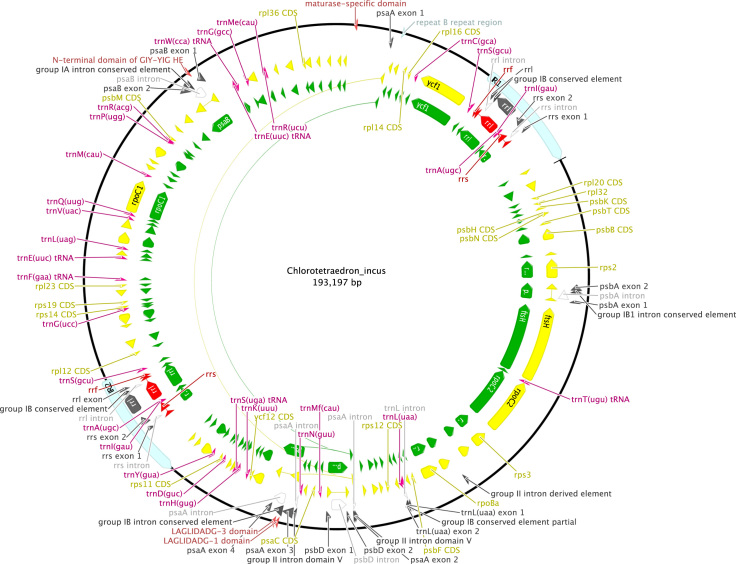
Schematic map of the chloroplast genome of *Chlorotetraedron incus* (KT199252, SAG 43.81).

**Fig. 6:**
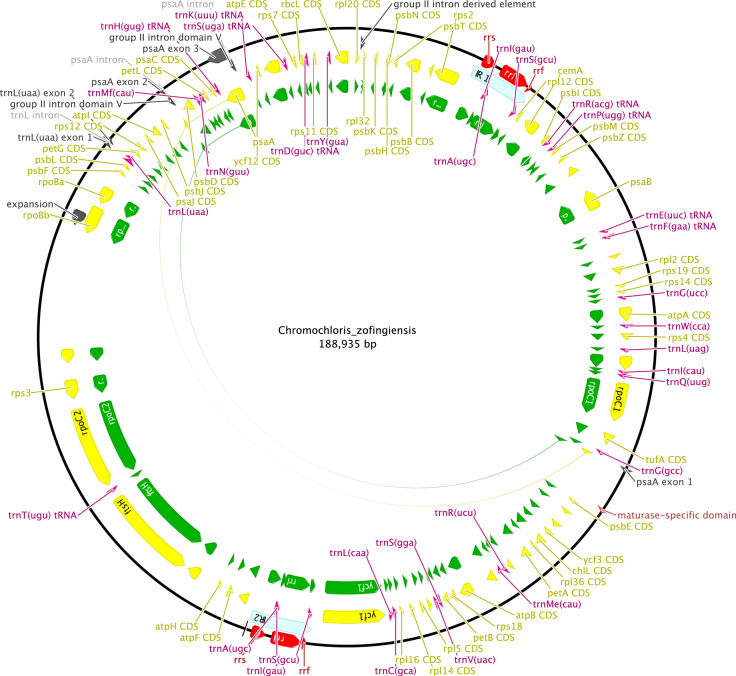
Schematic map of the chloroplast genome of *Chromochloris zofingiensis* (KT199251, UTEX 56).

**Fig. 7:**
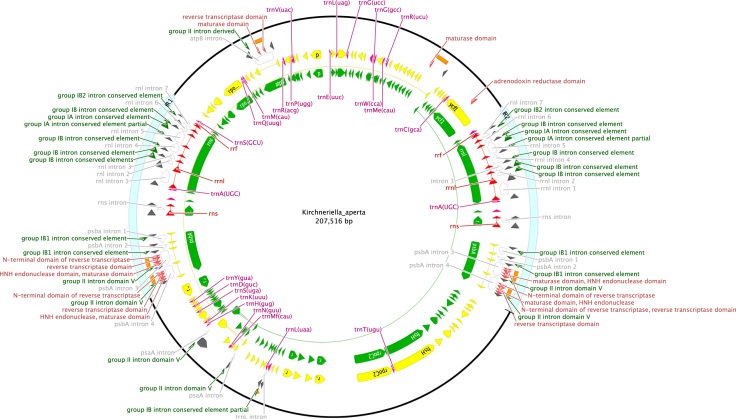
Schematic map of the chloroplast genome of *Kirchneriella aperta* (KT199250, SAG 2004).

**Fig. 8:**
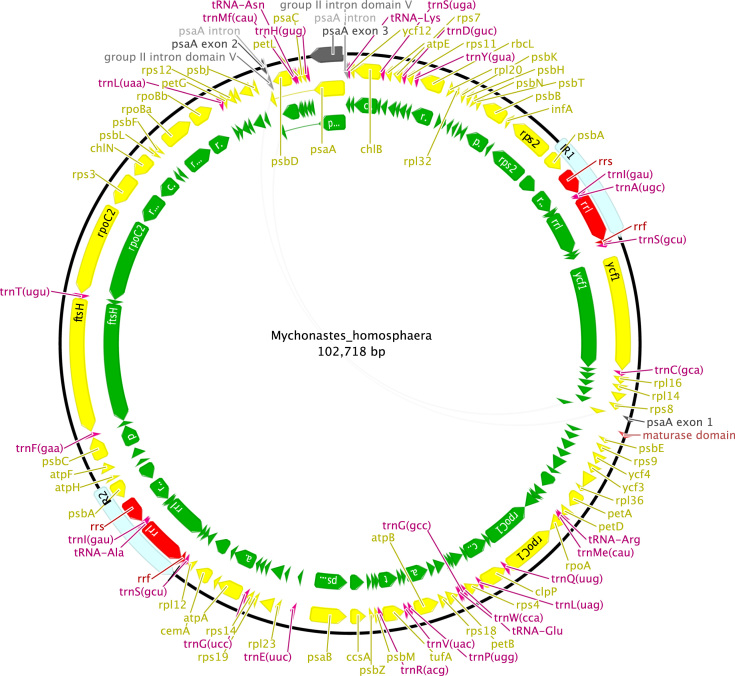
Schematic map of the chloroplast genome of *Mychonastes homosphaera* (KT199249, CAUP H 6502).

**Fig. 9:**
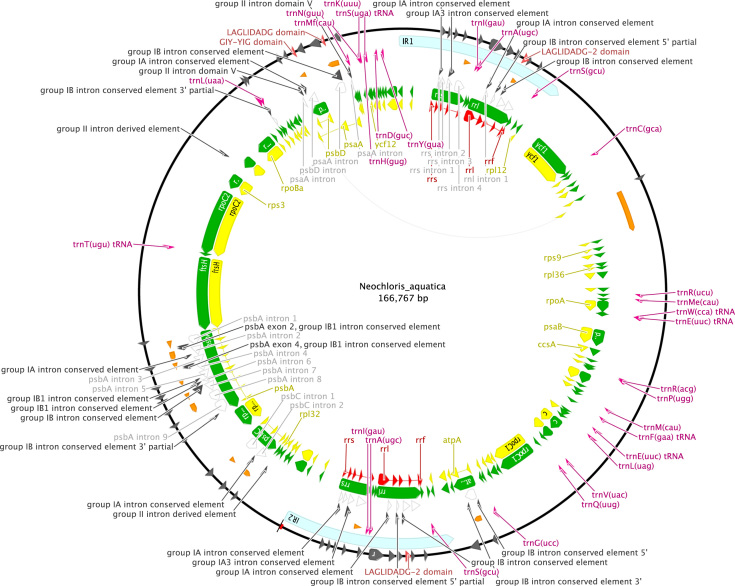
Schematic map of the chloroplast genome of *Neochloris aquatica* (KT199248, UTEX 138).

**Fig. 10:**
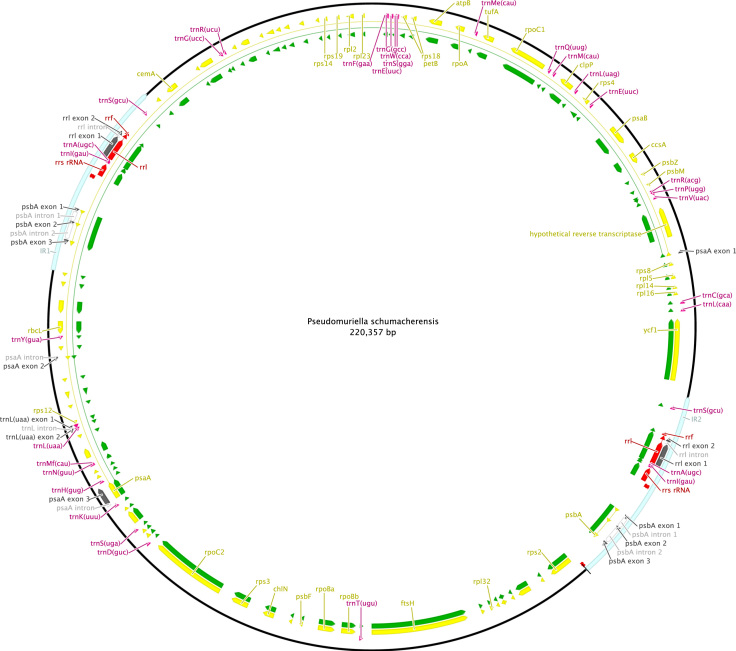
Schematic map of the chloroplast genome of *Pseudomuriella schumacherensis* (KT199256, SAG 2137).

**Fig. 11:**
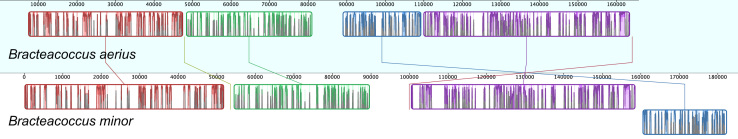
Analysis of synteny between the chloroplast genomes of two species of *Bracteacoccus*, *B. aerius* and *B. minor*, using MAUVE alignments. Colored and outlined blocks surround regions of the genome sequence of one genome that aligned to a corresponding part of the second genome, and lines connect blocks of putative homology. Within the blocks the colored bars indicate the level of sequence similarities.

**Fig. 12:**
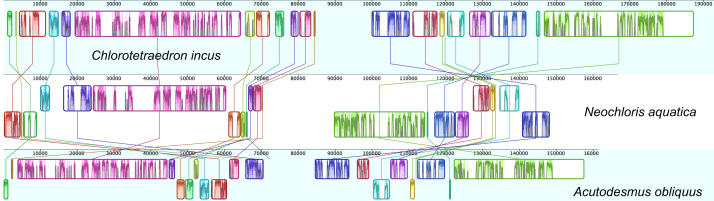
Synteny analysis among the chloroplast genomes of three species, *Acutodesmus obliquus*, *Neochloris aquatica*, and *Chlorotetraedron incus*. Colored and outlined blocks surround regions of the genome sequence that aligned to a corresponding part of the second genome, and lines connect blocks of putative homology. Within the blocks the colored bars indicate the level of sequence similarities.

**Fig. 13:**
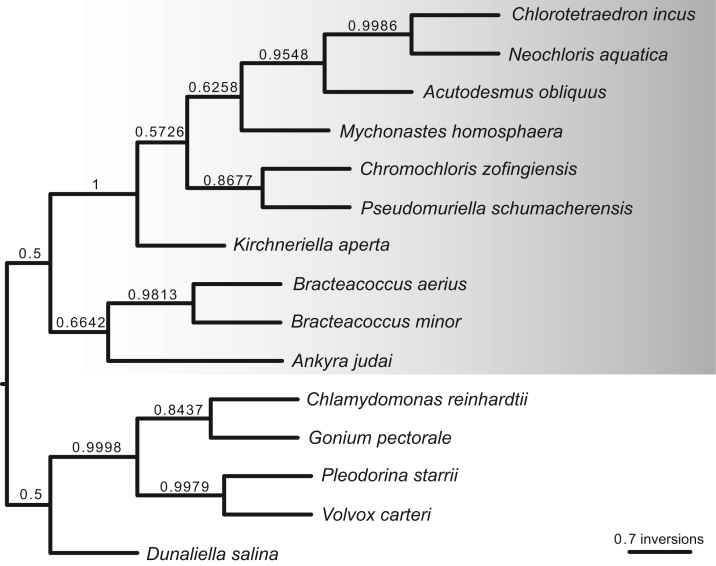
Badger phylogenetic tree inferred from gene order data present in completely sequenced chloroplast genomes of Sphaeropleales. Scale bar represents the number of inversions inferred to have occurred along a particular branch.

In this article we present chloroplast genome structural data for nine species of green algae (*GenBank: KT199248, GenBank: KT199249, GenBank: KT199250, GenBank: KT199251, GenBank: KT199252, GenBank: KT199253, GenBank: KT199254, GenBank: KT199255, GenBank: KT199256*; *NCBI:* http://www.ncbi.nlm.nih.gov), including comparison of gene and intron content (Fig. 1). Chloroplast genome maps with all annotated features are presented in Fig. 2, Fig. 3, Fig. 4, Fig. 5, Fig. 6, Fig. 7, Fig. 8, Fig. 9, Fig. 10. Synteny comparisons were performed for two sets of species (Fig. 11, Fig. 12). Gene order data were scored for 15 taxa in the class Chlorophyceae (GeneOrder.txt). A phylogenetic analysis of gene order using Badger is presented in (Fig. 13, GeneOrder.tre).

## 2 Experimental design, materials and methods

Full chloroplast genome sequences were obtained for 9 species of green algae in Sphaeropleales [1]. From these, genome maps were prepared in Geneious (version 6) (Fig. 2, Fig. 3, Fig. 4, Fig. 5, Fig. 6, Fig. 7, Fig. 8, Fig. 9, Fig. 10). Synteny maps were produced using the Mauve plugin in Geneious, which was also used to estimate the numbers of genomic rearrangements among taxa [2] (Fig. 11, Fig. 12). Gene order data were analyzed phylogenetically using Badger [3] for a total of 15 species in Chlorophyceae with fully sequenced cp genomes (Fig. 13). Only single-copy genes that were present in all included genomes (83 genes) were used for the analysis. Genes present in inverted repeats were counted once and inverted repeats were oriented to have the rRNA genes on the positive strand. For the trans-spliced *psaA* gene, only the first exon was considered. Badger was run for 10,000,000 generations, sampling every 100, with other settings set to default. The first 1000 samples were discarded as burnin.
