# Supplementary material for: Comparative analyses of chloroplast genome data representing nine green algae in Sphaeropleales (Chlorophyceae, Chlorophyta)
Source: Data Brief. 2016 Mar 9;7:558–70. doi: 10.1016/j.dib.2016.03.014 (PMC4802548; doi:10.1016/j.dib.2016.03.014)

**Conflict of Interest Form for manuscript DIB-D-16-00107**

**Title:** *Comparative analyses of chloroplast genome data representing nine green algae in Sphaeropleales (Chlorophyceae, Chlorophyta).*

**Authors:** Karolina Fučíková, Louise A. Lewis, and Paul O. Lewis

**Affiliations:** Department of Ecology and Evolutionary Biology, University of Connecticut, Storrs, CT, USA

**Contact email**: [Karolina.fucikova@gmail.com](mailto:Karolina.fucikova@gmail.com)

Conflicts of interest: none

Karolina Fucikova, corresponding author


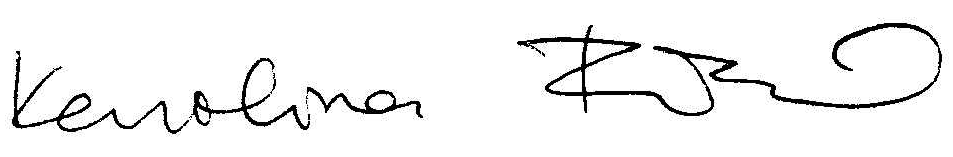

Supplement: Supplementary file 2 — Supplementary material [file mmc2.docx]
